# Investigation of antioxidant activities of *Pleurotus ostreatus* stored at different temperatures

**DOI:** 10.1002/fsn3.644

**Published:** 2018-04-16

**Authors:** Temelkan Bakir, Mertcan Karadeniz, Sabri Unal

**Affiliations:** ^1^ Department of Chemistry Faculty of Science and Letters Kastamonu University Kastamonu Turkey; ^2^ Department of Forest Engineering Faculty of Forestry Kastamonu University Kastamonu Turkey

**Keywords:** antioxidant capacity, different temperature, DPPH method, *Pleurotus ostreatus*

## Abstract

In this study, the antioxidant capacity of oyster mushroom (*Pleurotus ostreatus*) stored in five different temperature environments was investigated by DPPH (1,1 diphenyl‐2‐picryl hydrazyl) radical scavenging method. The antioxidant capacity results of oyster mushroom extracts were examined by spectroscopic measurements and expressed as percent of inhibition. The IC_50_ values of mushroom samples were calculated with DPPH method using calibration equations, and change in antioxidant properties was also tried to decipher by SEM images. The IC_50_ values of *Pleurotus ostreatus* for four different concentrations were also found in h_+20°C_ > inh_+4°C_> inh_−10°C_> inh_−20°C_> inh_−40°C_, respectively. As a conclusion, the temperature factor of oyster mushroom in the range of −40°C to +20°C storage conditions is directly proportional to the change of antioxidant properties. The results have shown the importance of logistics and storage conditions in preserving the antioxidant properties of oyster mushroom and similar food samples.

## 1 INTRODUCTION

Oyster mushrooms (*Pleurotus ostreatus*), which have a large economic share in the world among edible mushroom species, are actively used in medical treatments with antitumor, antibacterial, antiviral, and antioxidant properties. For this reason, storage conditions and shelf life are very important for biological activities such as in many food and consumption items. Antioxidants and phenolic compounds, especially in herbal food, are affected by storage conditions as well as by pH, temperature, and extraction conditions (Ruenroengklin et al., 2008). By determining the optimal temperature and storage conditions, it is possible to reduce the losses of different bioactive compounds and thus to increase the shelf life of foods.

Antioxidants are important compounds that can be produced by living organisms, as can be obtained from many natural sources, and protect their health by damping active oxygen and free radicals (Phachonpai, Wattanathorn, Muchimapura, Tong‐Un, & Preechagoon, 2010; Rahim, Salihon, Yusoff, Bakar, & Damanik, 2010; Tong‐Un, Muchimapura, Phachonpai, & Wattanathorn, 2010). Temperature is one of the most important factors affecting antioxidant activity. Generally, there is a decrease in the activity of antioxidants due to heating; however, changes in temperature may affect the mechanism of action of some antioxidants differently depending on the environment in which they are present (Réblová, 2012). In particular, studies in the lipid medium have shown that temperature is one of the most important factors determining the change of antioxidant activity (Evans, Kodali, & Addis, 2002; Kolb, Loyall, & Schafer, 2002; Marinova & Yanishlieva, 2003; Zhang, Wu, & Weng, 2004). However, according to our investigations, there are no studies observing the effects of antioxidant capacity change in mushrooms, especially at low temperatures.

Aim of this study is the determination of the changes in total antioxidant activities of *Pleurotus ostreatus* samples stored at different temperatures. In this study, therefore, percent of inhibition of mushroom samples were calculated with the help of calibration equation by using DPPH (1, 1 diphenyl‐2‐picryl hydrazyl) radical scavenging method. In addition, SEM imaging technique has been used to obtain information about the physical states of samples in each temperature environment with the aim of contributing to the study.

## 2 MATERIAL AND METHODS

All chemicals which were analytical grade provided from Sigma‐Aldrich Co. LLC. In each stage, deionized purity water was used. Absorbents were measured using a SHIMADZU the UVM‐1240 UV–Visible spectrophotometer (Shimadzu Corp., Kyoto, Japan manufactures) with a pair of identical quartz cuvette of 1 cm thickness at 517 nm. FEI brand, Quanta FEG 250 model scanning electron microscope (SEM) was used for morphology studies of mushroom specimens.

### 2.1 Mushroom identification

*Pleurotus ostreatus* mycelium (HK‐35) was bought from Agroma (Denizli, Turkey). Then, they cultivated in Kastamonu University Mushroom Research and Application Center Laboratory. The cultivated oyster mushrooms were identified morphologically by Prof. Dr. Sabri ÜNAL and kept with voucher number (370001) at Mushroom Research and Application Center of Kastamonu University.

### 2.2 *Pleurotus ostreatus*

Cap of mushrooms is an average of 15.6 cm wide; kidney‐shaped to fan‐shaped white to dark brown; sometimes fading slowly and becoming two‐toned. The gills are white and are attached to and running down the cap. The stipes are 4.3 cm long in average and white colored.

### 2.3 Preparation of mushroom extracts

Mushroom samples were stored for 24 hr at +20°C, +4°C, _*−*_10°C, _*−*_20°C and _*−*_40°C, and the samples were dried at 40°C for 48 hr. Each sample was pulverized, and then, mushroom extracts were prepared.

Twenty milliliter of 80% methanol was added to 2.5 g of dry mushroom and allowed to stand for 3 hr and then filtered. Then, 5 ml of 80% methanol was added to the dried mushroom and the mixture was allowed to stand for 2 hr and filtered again. A total of 25 ml of mushroom extract was obtained. The resulting mushroom extract was centrifuged at 5000 rpm and + 4°C for 10 min (Pedraza‐Chaverrí et al., 2004).

### 2.4 Preparation of DPPH calibration solutions

123 mg DPPH (1, 1‐diphenyl‐2‐picryl hydrazyl) was dissolved in 50 ml of absolute alcohol (6.25 × 10^−3^ mol/L). Then, diluted from this solution and different concentration (1.25 × 10^−3^ mol/L as well, 2.5 × 10^−4^ mol/L and 5 × 10^−5^ mol/L) of DPPH calibration solutions were prepared. The calibration graph is obtained by reading the absorbance value of the DPPH solutions. And, the calibration equation y = 7.62 × 10^3^c−0.018 (R^2^ = 0.999) was calculated for DPPH solutions at the concentration range of 5–25 × 10^−5^ mol/L.

### 2.5 Preparation of sample [Mushroom Extract + Ethanol + DPPH] system solution

The solution was prepared as follows: 3 ml (stock 1.9 × 10^−4^ mol/L) DPPH + X ml mushroom extract + (3‐X) ml of methanol (75%); total volume of 6 ml of the reaction mixture. (X = 0.05, 0.10, 0.15, 0.20 ml).

### 2.6 DPPH measurements

Antioxidant effects of mushroom extracts were performed by using DPPH method. Methanol solution of DPPH radical was purple and gives the maximum absorbance at 515–517 nm. As the antioxidant concentration increases, the color of the DPPH radical becomes lighter and thus the antioxidant concentration can be monitored by spectrophotometer. The amount of antioxidant which required to reduce DPPH concentration by 50% is a commonly used parameter to measure the antioxidant activity, and it is called IC_50_ (mg/ml) (Frankel & Meyer, 2000). In this study, different concentrated DPPH calibration solutions prepared with methanol incubated for 15 min at room temperature in the dark. Then, absorbance at 517 nm was recorded corresponding to the blank. In the same way, methanol‐DPPH solution which prepared for control was used as a standard.

Percentage of radical scavenging activity is calculated by the following formula:

$$\text{Inhibition},\;\;\%=[\left(C_{0}-C_{1}1\right)/C_{0}]\times 100$$

*C*_0_: Concentration of control solution (no antioxidant added) and *C*_1_: concentrations of sample solutions (when antioxidant was present) (Huang, Ou, & Prior, 2005).

The IC_50_ value was determined from the graph slope “y = mx + c” formula that obtained from the graph for standard Trolox and mushroom extracts (Mukherjee et al., 2011).

### 2.7 Statistical analysis

Relationship between antioxidant concentrations of mushrooms was calculated using descriptive statistical analysis with Microcal Origin Pro 8.5.1 (Origin Lab. Corp., Northampton, MA, USA). Statistically significant effects were investigated using SPSS software (SPSS Inc., Chicago, IL, USA) for Windows version 13.

## 3 RESULTS AND DISCUSSION

**Figure 1:**
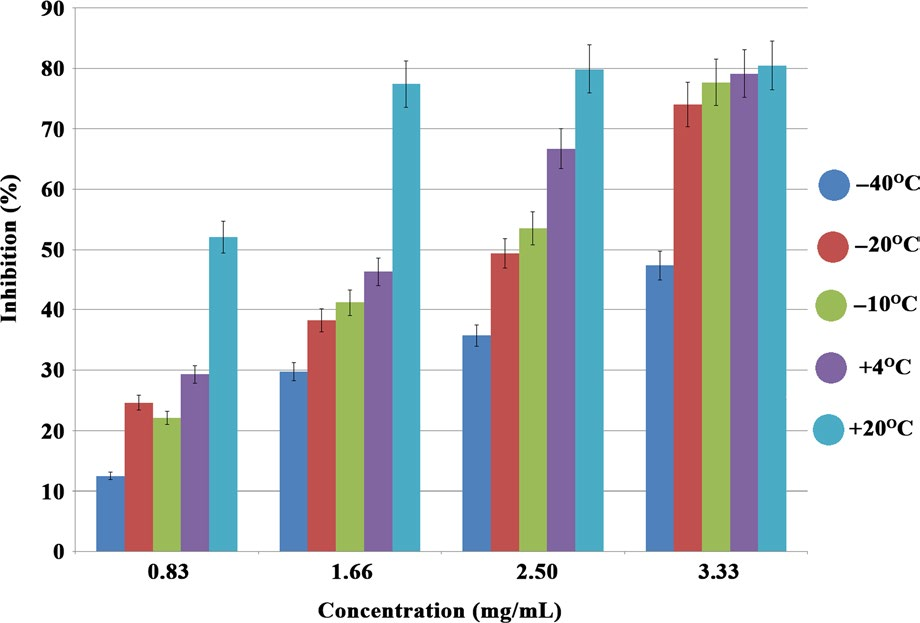
Changes in concentration and inhibition (%) of mushroom samples incubated at different temperatures (measured by DPPH assay). The calculated results are given as mean %95 confidence interval. The statistical significance was accepted at *p* < .05, (*n *= 4)

In this study, percent of inhibition of mushroom samples was calculated with the help of calibration equation using DPPH radical scavenging method. Accordingly, the antioxidant capacities of the mushroom species incubated at different temperatures are shown as percent of inhibition in Figure 1.

According to these results, it is obtained that different antioxidant properties for each mushroom samples. The findings of the inhibition (%) of *Pleurotus ostreatus* for four different concentrations were obtained inh_+20°C_ > inh_+4°C_> inh_*−*10°C_> inh_*−*20°C_> inh_*−*40°C,_ respectively. As can be seen, the lowering of the storage temperature of frozen food caused the antioxidant capacity to fall further. These results confirm the study of de Ancos, González, and Cano (2000). In addition, many researchers have shown that cold storage conditions in plant samples cause the reduction of antioxidant activity. (Oancea, Draghici, & Ketney, 2016; Sitthitrai, Ketthaisong, Lertrat, & Tangwongchai, 2015).

As shown in Figure 1, the difference between the inhibition percentages of mushroom samples incubated at different temperatures at high concentrations is reduced. However, as the storage temperature is lowered, the change in the percentage of inhibition and concentration is minimal. In addition, in some studies, the effect of drying temperature on the potency of polyphenols and antioxidant activity changes in plant extracts have been examined (Larrauri, Rupérez, & Saura‐Calixto, 1997).

In this study, the storage conditions were kept constant in all mushroom samples from time to time and the antioxidant activity change in four different concentrations of each mushroom sample was observed.

But we did not compare relation between phenolic antioxidants in the mushroom samples. Therefore, we are going to study polyphenolic contents of the mushroom species to make a definite judgment in another work.

As a result, IC_50_ values of mushroom samples that were calculated with DPPH method using calibration equations as shown in table 1. And they were found 3.486, 2.258, 2.144, 1.816 and 0.321 mg/ml for −40, −20, −10, +4, and +20 degree of celsius, respectively. Considering the IC_50_ values, mushrooms stored 1 day at room temperatures have more potent antioxidant activity compared to frozen samples at low temperatures.

**Table 1:** Absorbance values, concentration equations and IC_50_ values for of *Pleurotus ostreatus* stored at different temperatures

Temp. (^o^C)	Concentration (mg/ml)*	Absorbance (517 nm)	Concentration equation	*R* ^2^	IC_50_ (mg/ml)
	1.9 × 10^−4 ^mol/L DPPH 3 ml + Methanol (%75) 3 ml	0.682			
+20	0.83	0.327	y = 19.14c* + 43.86	0.809	0.321
	1.66	0.154			
	2.50	0.137			
	3.33	0.133			
+4	0.83	0.482	y = 20.38c* + 12.99	0.988	1.816
	1.66	0.366			
	2.50	0.227			
	3.33	0.142			
−10	0.83	0.531	y = 21.46c* + 4.00	0.977	2.144
	1.66	0.401			
	2.50	0.317			
	3.33	0.152			
−20	0.83	0.514	y = 19.11c* + 6.85	0.950	2.258
	1.66	0.421			
	2.50	0.345			
	3.33	0.177			
−40	0.83	0.597	y = 13.27c* + 3.74	0.946	3.486
	1.66	0.479			
	2.50	0.438			
	3.33	0.359			

Mushroom extract concentrations (*): 0.83; 1.66; 2.50; 3.33 mg/mL (g/L)

**Figure 2:**
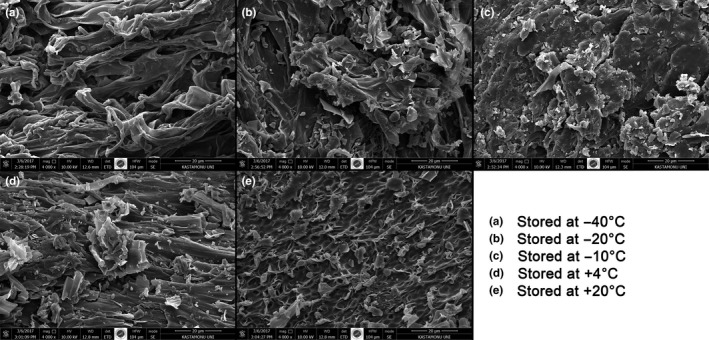
SEM images of *Pleurotus ostreatus* stored at different temperatures (a) −40°C, (b) −20°C, (c) −10°C, (d) +4°C, (e) +20°C

In this study, morphology of oyster mushrooms stored at different temperatures and then dehydrated by drying under the same conditions was analyzed by SEM images. As can be seen in the SEM image of Figure 2, it is more lumpy in the samples stored at −40°C and thus appears in a more open and accessible form in the external atmosphere. The structure of the samples at higher temperatures is particularly tight and integral with +20°C. For this reason, when bean mushrooms are kept in freezing conditions, it is seen that the structure is transformed from a compact and unusable form into an open and widely accessible form. This can help and accelerate the penetration of oxygen in the environment and the oxidation of antioxidant ingredients.

The changes in the macroscopic properties of the materials are due to the change in microstructure. For example, a porous structure may cause rapid water diffusion or rapid water loss during drying. On the contrary, a compact structure on the surface of the product may cause a slower moisture migration during drying. (Aguilera & Stanley, 1999; Xiao et al., 2009).Thus, microstructure investigations can help quantify product changes, particularly during the processing of food products, and can provide insight into mechanisms and changes in food texture changes (Deng & Zhao, 2008; Xiao & Gao, 2012).
